# Insights into the Flavor Profiles and Key Aroma-Active Compounds of Sichuan *Xiaoqu Qingxiangxing Baijiu* Across Distilling Stages

**DOI:** 10.3390/foods14162814

**Published:** 2025-08-14

**Authors:** Lei Tian, Pei Xu, Ji Qin, Guojun Hou, Qiao Huang, Ying Liu, Yu Li, Tongwei Guan

**Affiliations:** 1 Food Microbiology Key Laboratory of Sichuan Province, School of Food and Bioengineering, Xihua University, Chengdu 610039, China; tianlei@mail.xhu.edu.cn (L.T.);; 2 Department of Civil, Environmental, and Geo-Engineering, University of Minnesota, Minneapolis, MN 55455, USA; 3 Sichuan Langjiu Group Co., Ltd., Luzhou 646523, China; 4 Sichuan Tujiu Liquor Co., Ltd., Nanchong 637919, China

**Keywords:** Sichuan *Xiaoqu Qingxiangxing Baijiu*, distillation, odor-active compounds, electronic nose, electronic tongue, HS-SPME-GCxGC-MS

## Abstract

Distillation, a crucial step in *Baijiu* production, profoundly influences its flavor. However, the aroma-active compounds of Sichuan *Xiaoqu Qingxiangxing* (*SXQ*) Baijiu during distillation remain unclear. Here, we comprehensively analyzed the volatile flavor compound (VFC) composition and alcohol content variations across three key distillation stages (i.e., head, heart, and tail) of *SXQ Baijiu* using headspace solid-phase microextraction (HS-SPME) combined with comprehensive two-dimensional gas chromatography–mass spectrometry (GC×GC-MS), alongside electronic nose (E-nose) and electronic tongue (E-tongue). A total of 111 VFCs, 22 key odorants, and 11 pivotal differential VFCs were identified. Ethyl octanoate were identified as the most critical odor-active compounds, while isoamylol was recognized as a key difference marker. VFC concentrations in raw *Baijiu* decreased from head > heart > tail, while VFC categories increased from tail > head > heart. The flavor profiles of the head differ significantly from those of the heart and tail in *Baijiu* distillation. Alcohol content decreased as distillation proceeded. The E-nose and E-tongue effectively distinguished raw *Baijiu* samples from different distillation stages. This study enhances our understanding of volatile compounds and their aroma contributions during the distillation process of SXQ *Baijiu*. The findings provides bases for optimizing the distillation and quality-based classification of distillates in SXQ *Baijiu* production.

## 1. Introduction

*Baijiu* is a unique distilled liquor originating from China, produced from cereals through traditional solid-state fermentation, distillation, aging, and blending processes [1]. Among *Baijiu*’s primary aroma types, *Qingxiangxing* (*QXX*) *Baijiu* is characterized by a pure aroma, a soft and smooth taste, and a balance of sweetness and freshness, with ethyl acetate as its dominant aroma compound, complemented by ethyl lactate [1]. Sichuan *Xiaoqu Qingxiangxing* (*SXQ*) *Baijiu* have a distinctive flavor profile due to unique driving factors, including geographic environment, microclimate, fermentation starters (*Qu*), and production techniques. As a premium base liquor widely used in *Baijiu* blending, it demonstrates high consumer preference and substantial market potential.

Distillation is a critical step in *Baijiu* production, closely tied to yield and quality. Trace volatile flavor compounds (VFCs), such as alcohol and esters in fermented grains, are transferred into the raw *Baijiu* via steam. Owing to differences in boiling points and solubility, flavor compounds are distilled out sequentially during the distillation process [2], which results in distinct flavor profiles. The earliest distilled fraction (i.e., head) contains higher alcohol content but has a harsh and spicy flavor. In contrast, the middle distilled fraction (i.e., heart) offers a better overall taste with the highest quality. The last distilled fraction (i.e., tail) is characterized by a cloudy appearance and sour, astringent taste [3]. Compounds such as aldehydes in distilled raw *Baijiu* (especially at the beginning of distillation) may have adverse effects on human health. Therefore, in the brewing process, the head and tail of raw *Baijiu* are typically picked out and re-distilled based on these characteristics and accumulated experience, and the heart fraction of raw *Baijiu* is used for subsequent processing of *Baijiu* products due to its superior quality [2]. However, the composition of flavor compounds at different distillation stages remains insufficiently explored.

Currently, research on flavor changes during *Baijiu* distillation predominantly focuses on *Nongxiangxing* (*NXX*) *Baijiu* [2,4,5,6], whereas studies on *QXX Baijiu* (especially *SQX Baijiu*) are relatively scarce and only available in the Chinese literature [7,8,9,10]. The common findings in these studies were that ethyl acetate, acetaldehyde, and higher alcohols showed a gradual decline in the distillation process, and the content of acetic acid and ethyl lactate increased with distillation. However, these studies [7,8,9,10] primarily employed traditional gas chromatography (GC) methods to analyze compounds in *Baijiu* samples during the distillation process of *QXX Baijiu*, which presents significant limitations in identifying flavor compounds. Moreover, no studies have focused on the aroma-active compounds during the distillation process of *SQX Baijiu*.

Comprehensive two-dimensional gas chromatography–mass spectrometry (GC×GC-MS) offers significant advantages over traditional one-dimensional gas chromatography, particularly in peak capacity and resolution [11], which is essential for analyzing complex samples such as *Baijiu*. The electronic nose (E-nose) and electronic tongue (E-tongue), as artificial intelligence tools that simulate human olfactory and gustatory systems, can provide faster and more objective analyses than human sensory evaluation to facilitate the scientific classification of *Baijiu*. In addition, the aroma of *Baijiu* is primarily influenced by the aroma-active compounds it contains [12]. Revealing the flavor characteristics and aroma-active compounds of raw *Baijiu* at different distillation stages is crucial for understanding how raw *Baijiu* impacts the quality of the final *SQX Baijiu* product.

Therefore, in this study, a combination of electronic sensory analysis and headspace solid-phase microextraction (HS-SPME) coupled with GC×GC-MS was employed to investigate flavor variations and aroma-active compounds in raw *Baijiu* across three key distillation stages (head, heart, and tail) of *SQX Baijiu*. It aims to provide a scientific basis for quality-based fractionation selection and classification storage.

## 2. Materials and Methods

### 2.1. Samples Collection

The *SQX Baijiu* samples used in this study were collected from Sichuan Province, China. These raw *Baijiu* samples, distilled from fermented grains, were grouped into three groups of composite samples corresponding to the early, middle, and later distillation stages (i.e., head, heart, and tail). Glutinous red sorghum was used for brewing. The fermented grains were uniformly mixed with a small amount of rice husk and spread layer by layer into the Zengtong (a traditional Chinese steamer with 1.3 m diameter and 0.5 m height) to ensure loose and even distribution. Continuous and stable heating was maintained to ensure uniform upward penetration of steam. The steamer was immediately covered after loading. Once the water in the Zengtong boiled, distillation was carried out for approximately 26 min. During this period, the distillate flow rate was controlled (around 3 kg/min MPa), The distillate temperature is typically controlled within the range of 25–30 °C, and fresh liquor was collected from the condensation pipe. Professional distillery workers can preliminarily judge the alcohol content and quality by observing the shape, size, and duration of the liquor bubbles [13]. Following established production protocols, the entire distillation process was divided into three stages (head, heart, tail) to pick fractions for separate collection [5]. The head *Baijiu* refers to the initial distillate collected during the early distillation stage, consisting of a high-alcohol water mixture around 80% vol ethanol. The heart *Baijiu* was obtained after head removal until the ethanol concentration dropped to approximately 65% vol. Finally, the tail *Baijiu* comprises the late-stage distillate with reduced alcohol content around 45% vol. The samples were stored at 4 °C until further analysis.

### 2.2. Alcohol Content Measurement

The alcohol content was measured using an alcohol meter (CONJANT, Shanghai, China). By correcting the temperature of the raw *Baijiu* samples, the ethanol volume fraction was adjusted to reflect the alcohol content at 20 °C. Each sample was tested in triplicate.

### 2.3. E-Nose Analysis

The VFCs were detected using a PEN 3 E-nose (Airsense Inc., Schwerin, Germany). The E-nose consists of ten metal oxide sensors and pattern recognition software (Win Muster v.1.6.2) for data recording and analysis. Each sensor is cross-sensitive to different types of volatile compounds (Table 1). The raw *Baijiu* samples were diluted with ultrapure water at a 1:5 volume ratio. A 10 μL diluent was placed in a 10 mL bottle and equilibrated at room temperature (24 °C) for 10 min. The measurement conditions for raw *Baijiu* were as follows: sampling interval of 1 s, cleaning time of 90 s, reset time of 10 s, pre-injection time of 5 s, sample measurement time of 60 s, and sample flow rate of 300 mL/min. The system was cleaned (cleaning time lasted for 300 s) and standardized before and after each sample was determined. Each sample was measured in triplicate, and the average values were calculated as the final response value. The principal component analysis (PCA) and radar plot were used to analyze the E-nose data.

The E-nose is placed in an independent room with constant temperature and humidity and no obvious odors. Cosmetics, perfumes, and solvents with pungent odors cannot be used in the instrument room during measurements. The in situ calibration of electronic nose was carried out by setting relevant parameters through software in conjunction with the instrument’s built-in cleaning function before use. Clean air was filtered through activated carbon as the “zero gas” into the E-nose. During use, the resulting deviation can be evaluated by comparing the response values to “zero gas” before and after cleaning. If the sensor response line was not parallel to the baseline, the cleaning duration was extended. A relative deviation of ≤±10% between pre- and post-cleaning measurements indicates satisfactory cleaning efficacy and stable sensor performance.

### 2.4. E-Tongue Analysis

The E-tongue (Bosin, Shanghai, China) was used to analyze the taste characteristics of the raw *Baijiu* samples. The system consists of a sensor array, a signal regulation system, a test platform, and application software. The inert metals in the sensor array include platinum, gold, palladium, titanium, tungsten, and silver electrodes. Before data collection, the voltage was set at −1 to 1 V, and the sampling time was 120 s. The sensor detection head was cleaned with ultrapure water before and after each measurement. Briefly, 20 mL of the raw *Baijiu* sample was poured into a 50 mL beaker designated for E-tongue analysis. The samples were measured six times in parallel. The PCA was used to analyze the E-tongue data.

### 2.5. Extraction of VFCs by HS-SPME

Flavor compounds were extracted by HS-SPME. Briefly, 5 mL of the raw *Baijiu* sample and 1.5 g of NaCl were added to a 15 mL headspace flask. Subsequently, 20 μL of 2-octanol (0.0822 mg/mL) was added as an internal standard for VFC quantification. The sample was then preheated and equilibrated at 60 °C for 15 min. A 50/30 μm DVB/CAR/PDMS fiber (Supelco, Bellefonte, PA, USA) was inserted into the headspace to extract volatile compounds at 60 °C for 40 min. The fiber was then inserted into the GC injection port for desorption at 250 °C for 5 min.

### 2.6. VFC Detection in Raw Baijiu

The flavor compounds of distilled raw *Baijiu* were detected and analyzed using a GC×GC-MS instrument (GCMS QP2020 NX, Shimadzu, Kyoto, Japan). The one-dimensional column was a polar DB-WAX column (30 m × 0.25 mm × 0.25 μm), the two-dimensional column was a moderately polar DB-17MS column (1.2 m × 0.18 mm × 0.18 μm), and the modulation column was HV (C5–C30). Helium (99.999%) served as the carrier gas at a flow rate of 1 mL/min. The vaporization chamber was maintained at 250 °C for 9 min and 230 °C for 5 min, starting at 40 °C.

The temperature program was as follows: the initial temperature was 40 °C and held for 5 min, then raised to 230 °C at a rate of 5 °C/min and held constant for 9 min. The injector temperature was set at 250 °C. Mass spectrometry conditions were as follows: the electron energy was 70 eV, with an ion source and interface temperature of 200 °C and 280 °C, respectively. The mass scan ranged from 50 to 450 *m*/*z* with a scan speed of 0.2 s/scan. The solution of C_6_–C_30_ n-alkanes was analyzed using the same chromatographic conditions to calculate linear retention index based on retention times [14]. The flavor compounds were identified by comparing the mass spectra of the compounds with the standard mass spectra in the NIST20 library. The identification and quantification of volatile compounds referenced a previously reported method [12].

### 2.7. Identification of Differential VFCs

The orthogonal partial least squares discriminant analysis (OPLS-DA) and the variable importance in the projection (VIP) plot were used to evaluate differences in VFCs among distilled raw *Baijiu* samples from the three distillation stages. The key VFCs with significant differences were identified based on VIP ≥ 1 and *p* ≤ 0.05.

### 2.8. Relative Odor Activity Value (ROAV) Analysis

The ROAV is commonly used to evaluate the contribution of individual compounds to the overall aroma. The ROAV of each aroma compound was calculated according to Feng et al. [15]. The threshold values of aroma compounds were obtained by referencing the relevant literature (Table 2). The VFCs with ROAV ≥ 1 are regarded as key aroma compounds. The higher the ROAV, the greater the contribution to overall aroma. The partial least squares discriminant analysis (PLS-DA) was used to evaluate differences in ROAV among distilled raw *Baijiu* samples from the three distillation stages. The key ROAV with significant differences was identified based on VIP ≥ 1.

### 2.9. Statistical Analysis

The histogram, Venn diagram, and radar plots were created using Origin 7.0 software (OriginLab Corporation, Northampton, MA, USA). The PCA was used to examine inter-group variability and intra-group similarity among the samples. The raw data were log2-transformed and standardized using z-score (mean-centered and scaled by standard deviation) to ensure comparability across datasets. Correlation matrix was used for decomposition. Confidence ellipses were generated to visualize sample clustering patterns. The two-dimensional (2D) and three-dimensional (3D) PCA plots were conducted in https://www.omicstudio.cn/tool and https://bio-cloud.aptbiotech.com/plus/#/tools (accessed on 27 June 2025).

The one-way analysis of variance (ANOVA) was performed using GraphPad Prism software (Version 8.2.1, Inc., San Diego, CA, USA) to determine significant differences among raw *Baijiu* samples from different distillation stages. The OPLS-DA and PLS-DA were conducted using Simca 14.0 (Umetrics, Umeå, Sweden). The measurements were conducted in replicates (*n* ≥ 3). The results were presented as the average ± standard deviation (SD).

## 3. Results and Discussion

### 3.1. Alcohol Content Analysis

**Figure 1:**
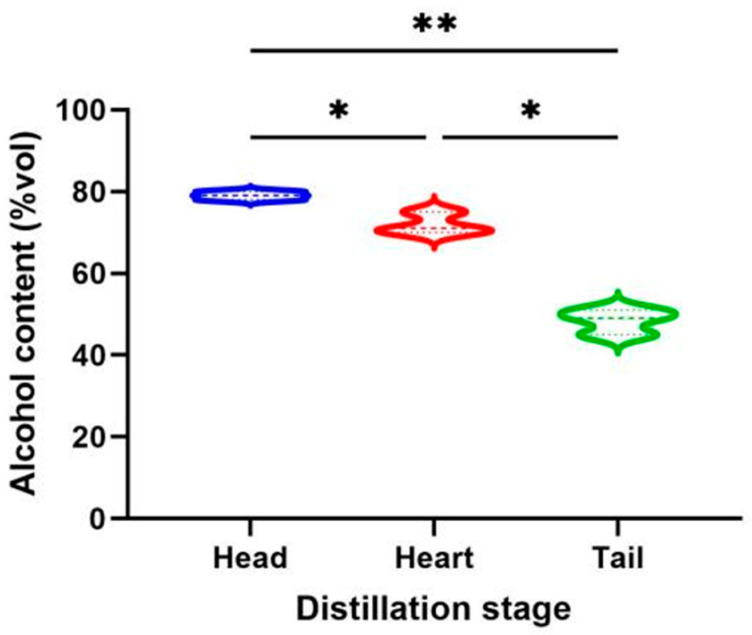
Alcohol content of raw *Baijiu* in different distillations stages. Note: * indicates *p* < 0.05, ** indicates *p* < 0.01.

The analysis of alcohol content is presented in Figure 1. The head *Baijiu* had the highest alcohol content at approximately 79% (*v*/*v*), followed by the heart *Baijiu* at 72% (*v*/*v*), and the tail *Baijiu* with the lowest at 49% (*v*/*v*). The results showed significant differences among the three distillation stages (Figure 1). The results of this study were compared with similar research on *Nongxiangxing Baijiu*, and we found that the alcohol content in our study was generally slightly higher than reported levels in other studies, particularly in the head and heart *Baijiu* [3,16]. This observation may be related to our relatively early initiation and late termination of liquor fraction collection during the distillation process. Currently, there are limited studies focusing specifically on the three critical stages (head, heart, and tail) [3,4,5,17]. Most studies have focused on the middle section of high-quality alcohol after the head and tail have been removed [8], or on the fraction obtained at different time or weight intervals [2,7,9,10,18,19,20]. The head and heart *Baijiu* showed good consistency with similar studies on *Qingxiangxing Baijiu*, while the tail *Baijiu* was slightly lower [8]. Most of the ethanol was distilled in large amounts during the early stage of distillation, likely due to ethanol’s low boiling point [7]. As distillation progressed, the alcohol content in various distillation stages gradually decreased. The head *Baijiu* shows extremely significant differences from the heart and the tail *Baijiu* (Figure 1). This further emphasizes the practical importance of stage-based fractionation in raw *Baijiu* production.

### 3.2. E-Nose Results Analysis

**Figure 2:**
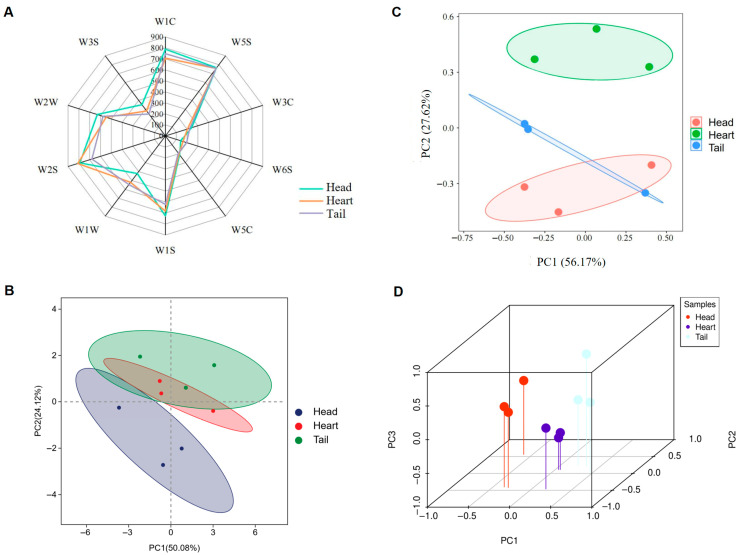
Overall volatile compound profile of raw *Baijiu* by E-nose and E-tongue measurement. (**A**) Radar plot of E-nose. (**B**) Principal component analysis of E-nose. (**C**) Two-dimensional principal component analysis of E-tongue. (**D**) Three-dimensional principal component analysis of E-tongue.

The E-nose data of raw *Baijiu* was analyzed by radar plot and PCA (Figure 2A,B) to evaluate the aroma characteristics. The PCA plot revealed that the head *Baijiu* showed partial separation from both the heart and tail *Baijiu*, while the heart and tail *Baijiu* exhibited relatively close distribution with partial overlap (Figure 2B). This suggests that the flavor profile of the head *Baijiu* differs significantly from the heart and tail *Baijiu*, whereas the latter two share greater similarity in flavor characteristics. The differences in flavor characteristics were reflected in the response values of seven sensors: W2S, W1C, W1S, W2W, W3S, W1W, and W6S (Figure 2A). This indicated that the content of alcohol and aromatic compounds, aromatic compounds, methane, aromatic and organic sulfides, long-chain alkanes, sulfur, and hydrides in the raw *Baijiu* of different stages varied considerably. The head and heart *Baijiu* showed the highest response value to the W2S sensor, and the value of heart *Baijiu* was slightly higher than that of head *Baijiu*. It indicated that the content of alcohol and aromatic compounds in VFCs of head and heart *Baijiu* was high. The tail *Baijiu* exhibited the highest response value to the W5S sensor. This indicated that the tail *Baijiu* VFCs detected by the E-nose sensor have a high level of nitrogen oxide. These results showed that the response values of raw *Baijiu* to each sensor were different, although their overall profiles were similar. This may be due to the raw *Baijiu* samples having similar volatile compounds with different concentrations. This is the first time that electronic nose was used to analyze raw *Baijiu* during the distillation process. Newly produced *Baijiu* contains compounds in an unstable equilibrium state [21], which may lead to suboptimal sensory attributes [22]. An aging process is required to mellow the flavor [23].

### 3.3. E-Tongue Results Analysis

The data obtained from the E-tongue were analyzed using 2D-PCA and 3D-PCA (Figure 2C,D) to explore the relationship between the overall taste profile and the raw *Baijiu* samples. The contribution rate of PC1 was 56.17%, while PC2 contributed 27.62%, resulting in a cumulative contribution rate of 83.79%. This indicated that the two principal components captured most of the information from the raw *Baijiu* samples, effectively reflecting the overall taste profile. The 3D-PCA output data are shown in Table S4. The samples exhibited distinct clustering in the 3D-PCA plot of the E-tongue, enabling clear differentiation among the distillation stages of the raw *Baijiu*. This finding demonstrated that the E-tongue effectively distinguished the flavor characteristics of the raw *Baijiu* samples.

### 3.4. VFC Analysis

**Figure 3:**
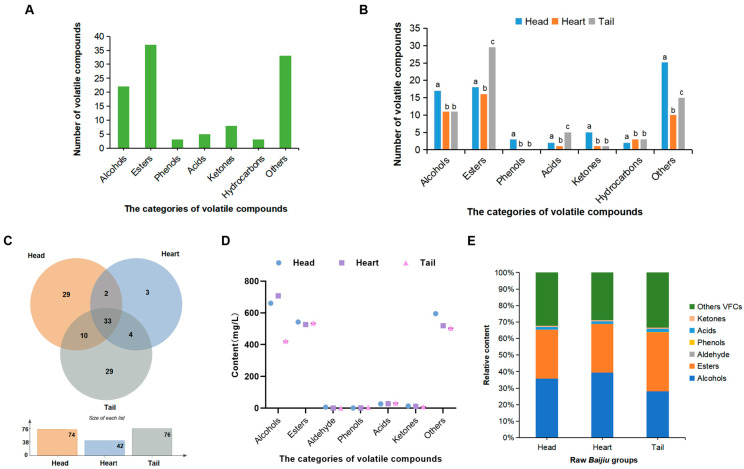
VFC analysis of raw *Baijiu* in different distillation stages. (**A**) Number of VFC categories in all raw *Baijiu*. (**B**) Number of VFC categories in head, heart, and tail *Baijiu*. (**C**) Venn diagram of VFC categories. (**D**) Content of VFCs in main categories across head, heart, and tail *Baijiu*. (**E**) VFC relative content in head, heart, and tail *Baijiu*. Different superscripts a–c indicate significant differences (*p* < 0.05).

A total of 111 flavor compounds were identified, comprising 22 alcohols, 37 esters, 3 aldehydes, 5 phenols, 8 acids, 3 ketones, and 32 other type of compounds (Table 2, Figure 3A). The head, heart, and tail stages of the raw *Baijiu* contained 75, 42, and 66 flavor compounds, respectively (Table 2, Figure 3B). It was evident that significant differences exist in the types of flavor substances between the head *Baijiu* versus both the heart and tail *Baijiu*, while the variations between the heart and tail *Baijiu* were primarily manifested in esters, acids, and other types (Figure 3B). Among these, 36 flavor compounds were common across all stages (Figure 3C), with esters being the most abundant (14), followed by alcohols (9) (Table S1). Furthermore, the head *Baijiu* contained 29 unique flavor compounds (Figure 3C), with hydrocarbons being the most prevalent, accounting for 15 compounds (Table S1). Three flavor compounds, including ethyl lactate, were exclusive to the heart *Baijiu* (Figure 3C). The tail *Baijiu* contained 28 unique flavor compounds (Figure 3C), with esters being the most abundant (15) (Table S1). The total amounts of VFCs in the head, heart, and tail *Baijiu* were 1847.05 mg/L, 1792.71 mg/L, and 1505.90 mg/L, respectively (Table 2). The overall concentration exhibited a trend of head > heart > tail *Baijiu* (Figure 3D, Table 2). This finding is consistent with the majority of studies on *NXX Baijiu* [3,5,6].

#### 3.4.1. Alcohols

The total content of alcohols (1791.17 mg/L) was the highest among all categories of flavor compounds in all distillation stages (Table 2), accounting for 29.44%, 39.49%, and 37.70% of the flavor compounds in the head, heart, and tail *Baijiu*, respectively (Figure 3E). The head *Baijiu* contained a greater variety of alcohols (17) compared to the heart and tail *Baijiu* (11) (Figure 3B, Table 2). Among the alcohols in the head *Baijiu*, 1-pentanol was the most abundant, approximately 212.53 mg/L, followed by 6-methyl-2-heptanol (150.90 mg/L) (Table 1). In the heart *Baijiu*, 1-propanol had the highest concentration (208.96 mg/L), followed by 1-pentanol (205.89 mg/L) and ethanol (113.86 mg/L) (Table 2). Notably, both the ethanol concentration (113.86 mg/L) and the 1-propanol concentration (212.77 mg/L) were the highest among the three stages (Table 2), showing an initial increase followed by a decline during the distillation. This indicated that the middle stage played a significant role in influencing the overall *Baijiu* yield. In the tail *Baijiu*, isoamylol was the most abundant (212.77 mg/L) (Table 2), substantially surpassing other compounds and serving as the primary flavor compounds of this stage. The concentrations of 1-dodecanol and 1-pentanol exhibited a gradual decrease from the head to the tail *Baijiu*, while the levels of 1-hexanol, phenylethyl alcohol, and isoamylol exhibited a corresponding increase. These compounds predominantly consisted of higher alcohols, which were the main constituents of the distilled raw *Baijiu*. 1-Pentanol is characterized by the aromatic qualities associated with fermented grains [24]. 1-Hexanol primarily exhibits floral and fruity [18]. Phenylethyl alcohol presents a flowery odor, characterized by its soft and enduring scent [25]. Higher alcohols play a crucial role in enhancing the bouquet of esters, thereby enriching the overall aroma and imparting a lingering finish to the *Baijiu* [26]. They are indispensable components of *Baijiu*, serving as primary sources of sweetness enhancement. However, excessive concentrations can lead to an unpleasant bitter off-flavor, known as the “fusel oil taste” [27]. The high levels of higher alcohols, particularly 1-pentanol, 1-propanol, isobutanol, and isoamylol, in the head and tail *Baijiu* may contribute to a pungent and harsh taste, creating an unpleasant sensory experience. As the distillation process progressed, the total alcohol content initially increased and then declined. This is consistent with Liu et al.’s study [28] on the variation in flavor components in the distillation process of *NXX Baijiu*. This phenomenon may be attributed to the low molecular weight and boiling points of most alcohols, which tend to evaporate alongside ethanol during the early stage of distillation, followed by a gradual reduction in the later stage. The high content of isoamyl alcohol is one of the characteristics of *SXQ Baijiu*, particularly in the early distillation fractions. When implementing the quality-based selective distillation process to enhance ethyl acetate content, careful consideration should be given to controlling the levels of isobutanol and isoamyl alcohol.

Remarkably, methanol was not detected in the head *Baijiu*, and furfural was not detected in the tail of the liquor. Only 5-Hydroxymethylfurfural (5-HMF) was detected in the head *Baijiu*, and the content was extremely low. Furfural and 5-furfural often coexist together. During the fermentation process involving bran shells, harmful compounds such as furfural and methanol may be produced [29]. Currently, there are no national standards specifying the minimum limits for 5-HMF and furfuraldehyde in the production of *Baijiu*. We have consulted the relevant literature, and it has been shown that the content of 5-HMF in six types of *Baijiu* (including *Nongxiangxing Baijiu*, *Jiangxiangxing Baijiu*, *Qingxiangxing Baijiu*, *Fengxiangxing Baijiu*, *Zhimaxiangxing Baijiu*, and *Jianxiangxing Baijiu*) is approximately 0.03 to 0.29 μg/mL [30]. Research has proven that among the three major aroma types of *Baijiu* (*Jiangxiangxing Baijiu*, *Nongxiangxing Baijiu*, and *Qingxiangxing Baijiu*), the content of furfural in clear-aroma *Baijiu* is the lowest [31]. It is reported that the furfural content in Fenjiu is approximately 3 to 4 mg/L, and the furfural content in other clear-aroma *Baijiu* types is also below 10 mg/L [31]. The 5-HMF and furfuraldehyde content in *Baijiu* is mainly generated through reactions such as thermal degradation of sugar and other substances during the brewing process [32]. Its content is affected by various factors such as raw materials, brewing techniques, and distillation conditions. The absence or low concentrations detected in our research results may be attributed to the substantial reduction in rice husk usage through innovative processing techniques, combined with optimized low-temperature fermentation and precise control of distillation temperature and duration. This may indicate that the manufacturers of the samples used in our study have relatively superior production processes, which effectively reduces the generation of furfural substances.

#### 3.4.2. Esters

Ester is one of the primary sources of aroma in Chinese *Baijiu*, imparting characteristics such as fragrance, freshness, and richness [33]. They mitigate the harshness of *Baijiu*, resulting in a softer and harmonious overall palate. Furthermore, esters enhance the smoothness of *Baijiu* [34]. The results indicated that the quantity of esters (37) in the raw *Baijiu* exceeded that of alcohols (22) (Figure 3B), although the concentration of esters (1604.31 mg/L) was slightly lower than that of alcohols (1791.17 mg/L) (Table 2). The relative content of ester compounds in the head, heart, and tail *Baijiu* was 24.17%, 29.36%, and 47.81%, respectively (Figure 3E). The content and quantity of esters (Figure 3B, Table 2) in the tail *Baijiu* were much higher than that in the head and heart *Baijiu*. These results were consistent with the trends observed by Ding et al. [4] in their study of *NXX Baijiu*. The substantial accumulation of esters in the tails *Baijiu* contribute to an exceptionally rich aroma. The ethyl acetate was the highest in the head and tail *Baijiu* with 140.34 mg/L and 135.46 mg/L, respectively (Table 2), followed by ethyl caprylate, ethyl caprate, ethyl laurate, ethyl hexanoate, ethyl oleate, phenethyl acetate, and ethyl linoleate. In the heart *Baijiu*, ethyl palmitate had the highest concentration (128.57 mg/L), followed closely by ethyl acetate (127.37 mg/L) (Table 2). These findings indicated that ethyl esters are the primary contributors to the ester aroma of *QXX Baijiu*, accounting for more than 90.18% of the total ester content in the raw *Baijiu* (Table 2). Ethyl esters are synthesized through the condensation of acyl-CoA and ethanol, catalyzed by ethyl ester biosynthesis (Eeb1p) and ethanol hexanoyl transferase (Eht1p) [35]. These compounds impart an elegant and sweet aroma to the *Baijiu*. Ethyl palmitate, a high-molecular-weight and high-boiling-point fatty acid ester, is characterized by a waxy scent and a sweet, rich flavor with a prolonged aftertaste. It plays a critical role in enhancing the stability and mouthfeel of *Baijiu*, contributing to mitigating astringency [36]. However, excessive ethyl palmitate may impart an oily sensation or cause cloudiness in low-alcohol *Baijiu* [37]. Ethyl acetate and ethyl butyrate were more abundant in the head *Baijiu* than in the heart and tail *Baijiu*. The variation pattern of ethyl acetate is consistent with findings reported in other literature on *QXX Baijiu* [5,9,10]. This may be attributed to the high solubility of these substances in ethanol, which evaporates in large quantities along with these compounds during the early stages of distillation. As the distillation process progresses and ethanol concentration decreases, the levels of ethanol-soluble compounds also decline. Ethyl acetate serves as the dominant aromatic component in *SXQ Baijiu*, exhibiting its highest concentration during the initial distillation phase. Its content significantly influences the *Baijiu*’s stylistic characteristics. Butyl octanoate and isoamyl decanoate were detected only in the tail *Baijiu*, while ethyl lactate was exclusive to the heart *Baijiu*. Phenethyl acetate decreased across all three stages, whereas diethyl succinate and ethyl laurate gradually increased. Ethyl lactate has a weak aroma and a slightly sweet taste, which enhances the smoothness and sweetness of *Baijiu* while extending its aftertaste due to its non-volatility [38]. This is crucial for maintaining the integrity of the *Baijiu* style [39]. Phenethyl acetate, reported as a key contributor to the aroma of *Xiaoqu Baijiu*, is likely formed through the esterification of acetic acid and phenylethyl alcohol [40]. It is characterized by floral and fruity, described as “banana and apple”. Diethyl succinate and ethyl laurate showed an increasing trend with the prolongation of distillation time. This may be because these compounds have a higher boiling point and gradually evaporate and accumulate in the tails.

#### 3.4.3. Acids

The head, heart, and tail *Baijiu* contained 5, 1, and 4 major acids, respectively (Figure 3B), with significant variations in their composition, and the relative contents were 1.21%, 1.53%, and 2.81%, respectively (Figure 3E). Acetic acid was the most abundant acid in all three stages, with concentrations of 23.03 mg/L, 27.46 mg/L, and 30.03 mg/L in the head (Table 2), heart, and tail *Baijiu*, respectively. It showed a gradual increase and then reached its highest level in the tail *Baijiu*. This indicated that acetic acid is the primary acid in the raw *Baijiu*. As a key precursor for ester synthesis [41], acetic acid contributes to the flavor formation of *SXQ Baijiu* and significantly influences the yield and quality of the final product. 1,2-Benzenedicarboxylic acid, linoleic acid, hexanoic acid, and octanoic acid were detected exclusively in the head *Baijiu*, which contained a greater variety of acids compared to the heart and tail *Baijiu*. As the distillation process progressed, the total acid content exhibited an overall increasing trend. These findings are consistent with previous reports on the changes in volatile aromatic compounds during the distillation of *Baijiu* [3,9,19,42].

#### 3.4.4. Aldehydes

Notably, aldehydes were exclusively detected in the head *Baijiu* in our study, with the relative contents being 1.21% (Figure 3E). This finding differs from the increasing trend of furfural compounds observed during distillation in other studies on *QXX* and *NXX Baijiu* fractions [5,43]. The concentrations ranked in descending order as acetaldehyde ethyl amyl acetal, L-glyceraldehyde, and 5-hydroxymethylfurfural (Table 2). Due to the generally lower boiling points of aldehydes compared to acids, alcohols, and esters, they are distilled earlier along with ethanol. Excessive levels of aldehydes can accumulate in the human body, leading to a prolonged metabolic cycle and significant health risks [44]. Additionally, high aldehyde content can result in a rough and harsh taste in the *Baijiu*. It was evident that the aldehyde content in the head *Baijiu* is extremely high; therefore, they are generally removed in the production [20]. Furthermore, the distilled raw *Baijiu* often requires storage to reduce its harshness, promote balance, and develop a smoother taste.

In this study, the content of furfural was not detected, and the content of 5-hydroxymethylfurfural (5-HMF) was extremely low (Table 2). As we all know, furfural and (5-HMF) often coexist together. The main sources of furfural are bran, sorghum husk, and wheat straw combined with fermented grains [29]. Currently, there are no national standards specifying the minimum limits for furfural and (5-HMF) in the production of *Baijiu*. It has been shown that the content of 5-HMF in six types of *Baijiu* (including *Nongxiangxing Baijiu*, *Jiangxiangxing Baijiu*, *Qingxiangxing Baijiu*, *Fengxiangxing Baijiu*, *Zhimaxiangxing Baijiu*, and *Jianxiangxing Baijiu*) is approximately 0.03 to 0.29 μg/mL [30]. Research has proven that among the three major aroma types of *Baijiu* (*Jiangxiangxing Baijiu*, *Nongxiangxing Baijiu*, and *Qingxiangxing Baijiu*), the content of furfural in clear-aroma *Baijiu* is the lowest [31]. It was reported that the furfural content in Fenjiu is approximately 3 to 4 mg/L, and the furfural content in other *Baijiu* types is also below 10 mg/L [31]. The 5-HMF and furfural content in *Baijiu* is mainly generated through reactions such as thermal degradation of sugar and other substances during the brewing process [32]. Its content is affected by various factors such as raw materials, brewing techniques, and distillation conditions. The absence or low concentrations detected in our research results may be attributed to the substantial reduction in rice husk usage through innovative processing techniques, combined with optimized low-temperature fermentation and precise control of distillation temperature and duration. On the one hand, this indicated that the manufacturers of the samples used in our study have relatively superior production techniques, which effectively reduces the generation of furfural substances.

#### 3.4.5. Phenolic Compounds

The head, heart, and tail *Baijiu* contained two, one, and five types of major phenolic compounds (Figure 3A), with concentrations of 1.29 mg/L, 0.91 mg/L, and 4.94 mg/L, respectively (Table 2). And the relative contents were 1.21%, 1.53% and 2.81% in head, heart, and tail *Baijiu*, respectively (Figure 3E). This variation trend differs from that of *NXX Baijiu* [44], which first increases and then decreases. The tail *Baijiu* had the greatest variety and highest concentration of phenols. Phenol exhibited the highest concentration in the tail *Baijiu* (Table 2), while 4-tert-octylphenol showed the highest concentration in the head *Baijiu*. Phenolic compounds, with high boiling points, predominantly impart smoky and medicinal flavors [45]. Phenolic compounds significantly contribute to the aroma, taste, and stability of *Baijiu*. Additionally, phenolic compounds play crucial roles in health, offering antioxidant, anti-tumor, and immune-boosting properties [46].

#### 3.4.6. Ketones

The head, heart, and tail *Baijiu* contained two, three, and three types of major ketones, respectively (Figure 3A). The head *Baijiu* exhibited the fewest varieties of ketonic compounds, consisting solely of 2-nonanone and 2-octanone, while the heart and tail *Baijiu* also included acetoin. The total concentrations of ketones in the head, heart, and tail *Baijiu* were 13.63 mg/L, 11.22 mg/L, and 8.44 mg/L, respectively (Table 2), and the relative contents were 0.74%, 0.63%, and 0.56% (Figure 3E). The head *Baijiu* exhibited the highest ketones concentration. Among these, 2-octanone had the highest concentration across all stages. The 2-nonanone is characterized by the aroma of roasted wheat and fresh grass [47]. This differs from the characteristics of the highest ketones in the middle section of *NXX Baijiu* [4]. Acetoin, with a buttery aroma, enhances the overall richness of *Baijiu*, contributes to a harmonious mouthfeel, and provides an enduring aftertaste [12].

In summary, our study identified far more flavor substances than the reported *QXX Baijiu*, approximately three times the number of reported substances, providing deeper insights into its flavor profile. Notably, we observed unique characteristics distinct from other *QXX Baijiu*, particularly the absence of aldehydes in the heart and tail fractions, which suggests potential safety advantages and subsequent utilization value. Compared with other flavors of *Baijiu*, it was found that most acids exhibited an increasing concentration during distillation, and esters represented by ethyl acetate generally decreased. However, the specific variation patterns of individual compounds differed across studies, showing the complexity of compound-specific distillation.

### 3.5. Potential Differential Marker of VFCs

**Figure 4:**
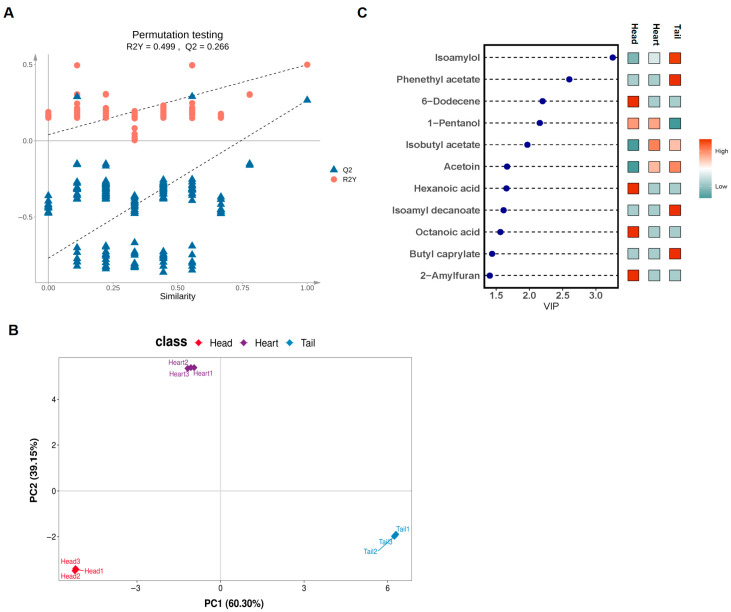
OPLS-DA analysis of the VFCs in head, heart, and tail *Baijiu*: (**A**) permutation test, (**B**) scores, and (**C**) VIP plot.

**Figure 5:**
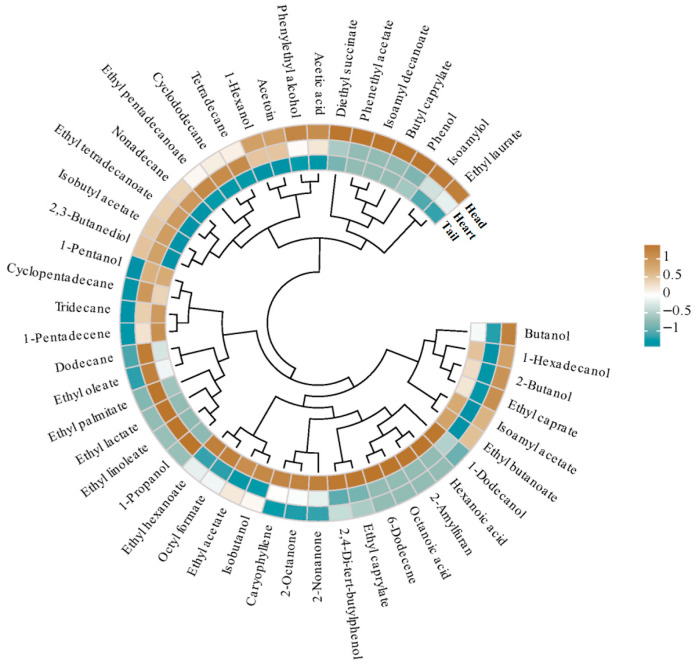
The heatmap of the VFCs in head, heart, and tail *Baijiu*.

Multivariate analysis was performed using OPLS-DA to better differentiate raw *Baijiu* and identify potential biomarkers (Figure 4A). The PCA analyses were conducted based on the quantitative untargeted omics data (Figure 4B). All raw Baijiu samples were distinct into three groups (Figure 4B). The aromatic profiles of the different stages varied distinctly (Figure 5). The VIP values were used to analyze the differences in VFCs across different distillation stages, further exploring the impact of the distillation process on the VFCs of raw *Baijiu*. A total of 11 differential VFCs with VIP > 1.0 and *p* < 0.05 were identified, including 2 alcohols, 4 esters, and 2 acids (Table S2, Figure 4C). Notably, isoamylol had the highest VIP value (3.25), followed by phenethyl acetate (2.60), (Z)-6-dodecene (2.20), and 1-pentanol (2.16) (Table S2). Furthermore, several esters and alcohols, including hexanoic acid, octanoic acid, isoamyl decanoate, butyl caprylate showed significant differences.

Isoamylol is the main higher alcohol in *Baijiu*, which contributes to flavor and quality of *Baijiu*. The appropriate amount of isoamylol can make the *Baijiu* mellow, and give it an elegant aroma. If the content of isoamylol in *Baijiu* is too high, it will make the *Baijiu* taste bitter and astringent. Isoamylol takes longer to oxidize in the human body than ethanol, so isoamylol remains in the human body for a long time. This causes intoxication, headaches, dizziness, and other adverse symptoms. Moderate reduction and control of isoamylol content in *Baijiu* can improve the drinking comfort.

In summary, our study identified far more flavor compounds than reported in the literature [7,8,9,10], about three times the number of reported compounds, and provided a more in-depth analysis of the flavor of *QXX Baijiu*.

### 3.6. Analysis of ROAV Value

**Figure 6:**
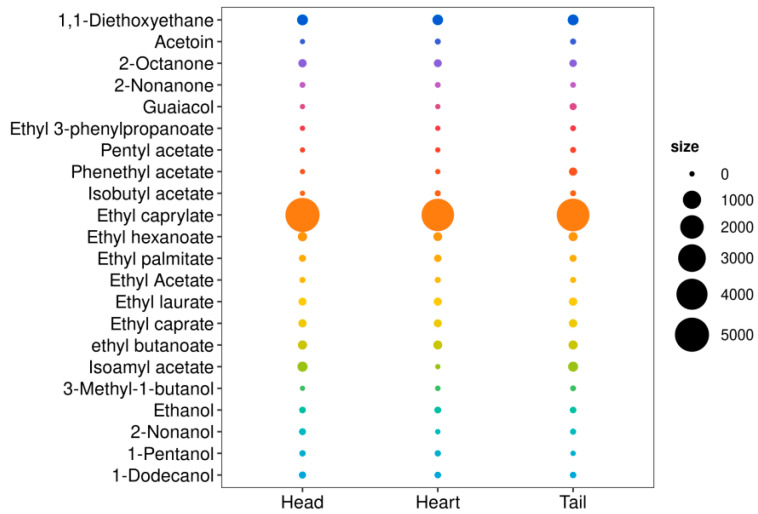
Bubble plot of volatile compounds with OAV ≥ 1 in head, heart, and tail *Baijiu*.

The ROAV is defined as the ratio of the concentration of a flavor compound in the *Baijiu* to its odor threshold, reflecting the compound’s contribution to the overall flavor profile [38,48]. Generally, a compound is considered to have a significant impact on flavor only when its ROAV exceeds 1. A total of 22 aroma-active compounds with ROAV > 1 were identified in this study, including 12 esters, 5 alcohols, 3 ketones, 1 alkane, and 1 phenol (Figure 6). In the head, heart, and tail *Baijiu*, 16, 16, and 20 aroma-active compounds with ROAV > 1 were detected, respectively (Table 3). Notably, 11 aroma-active compounds consistently exhibited ROAV > 1 in raw *Baijiu* across all three stages, including 2-nonanone, 1-dodecanol, ethanol, ethyl caprate, ethyl laurate, ethyl acetate, ethyl palmitate, ethyl hexanoate, ethyl caprylate, 2-octanone, and 1,1-diethoxyethane (Table 3). Among all the aroma-active compounds, ethyl caprylate exhibited the highest ROAV value (4464.37–5020.35) (Table 3, Figure 6), contributing sweet and pineapple-like fruity aromas. It was followed by 1,1-diethoxyethane with floral and fruity (169.24–186.95), ethyl hexanoate characterized by fruity and sweet aromas (86.27–100.31), as well as isoamyl acetate with banana-like and sweet aromas (136.09–143.7) (Table S3) [49].

**Figure 7:**
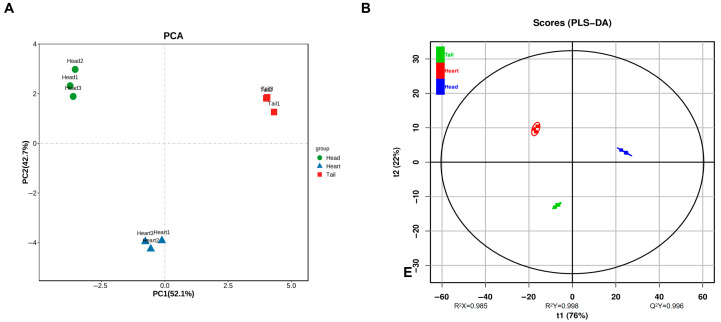
PLS-DA analysis of aroma-active compounds in head, heart, and tail *Baijiu*: (**A**) principal component analysis; (**B**) scores.

Considerable variation was observed in the ROAV values of aroma-active compounds across different distillation stages of the raw *Baijiu*. In the head *Baijiu*, compounds such as ethyl caprylate, 1,1-diethoxyethane, isoamyl acetate, ethyl hexanoate, and ethyl butyrate showed high ROAV values of 5020.35, 186.95, 143.7, 100.31, and 100.31, respectively (Table 3). These high-ROAV compounds were predominantly low-molecular-weight, highly volatile esters, which contributed intense fruity and sweet aromas to *Baijiu* [60]. In the heart *Baijiu*, ethyl caprylate and 1,1-diethoxyethane had high ROAV values of 4464.37 and 169.24, respectively (Table 3). In the tail *Baijiu*, ethyl caprylate, 1,1-diethoxyethane, and isoamyl acetate exhibited high ROAV values of 4541.27, 184.78, and 136.09, respectively (Table 3). To further investigate inter-group differences and category prediction among samples, PLS-DA was employed (Figure 7A,B and Figure S1A,B). The model parameters were validated through permutation testing (Figure 7A). The loading plot (Figure S1B) and score plot (Figure 7B) were combined to illustrate the influence of each variable in discriminating the difference between groups. The VIP values were used to evaluate the significance of variables in the PLS-DA model. The results showed that four compounds with VIP scores >1 (Table S3). Ethyl caprylate and isoamyl acetate had higher VIP values of 2.936 and 2.229, respectively, followed by phenethyl acetate (1.685), guaiacol (1.010) (Table S3). Higher VIP values indicate greater importance of these aroma compounds in the discrimination and difference analysis of raw *Baijiu* samples. These findings demonstrate that these four compounds contribute significantly to *Baijiu* aroma. Guaiacol, with a smoky and aromatic flavor, not only enhances the aroma of *Baijiu,* but also enhancing human immunity [1]. Ethyl caprylate played the most significant roles across all three distillation stages, showing the characteristics of head > tail > heart, greatly influencing the aroma profile of the raw *Baijiu*. Although the absolute concentration of ethyl caprylate detected was not particularly high, its ROAV contribution far exceeded those of traditionally recognized key flavor compounds in *QXX Baijiu*, such as ethyl acetate and ethyl lactate. This highlights its importance as a critical aroma-contributing compound in *SQX* raw *Baijiu*.

Ethyl hexanoate, as a primary aroma compound in *QXX Baijiu*, is primarily synthesized through the esterification of hexanoic acid and ethanol [61]. It is characterized by a pineapple-like aroma, enhancing the richness and fullness of the *Baijiu* [58]. Isoamyl acetate, with a strong fruity aroma [58], is a key component that determines the aroma and character of *Baijiu*. Appropriately increasing the concentrations of ethyl acetate and isoamyl acetate in *Baijiu* can help harmonize and balance its flavor. Ethyl acetate and ethyl butyrate provide typical green apple, strawberry, pineapple, and sweet aromas. When the ROAV values of ethyl acetate, ethyl butyrate, and ethyl hexanoate exceed 1, they likely contribute significantly to the overall aroma of *Baijiu* [58]. While 1-pentanol and 1,1-diethoxyethane have high absolute concentrations, their ROAV values were relatively low. Conversely, isoamyl acetate, ethyl hexanoate, and ethyl caprylate exhibited relatively low absolute concentrations but high ROAV values. Assessed from both concentration and threshold perspectives, the ROAV elucidates the contributions of aroma components to the overall flavor system, offering an effective technical approach for characterizing key aroma compounds in *Baijiu*.

### 3.7. Correlation Between E-Nose, E-Tongue, Alcohol Content, and HS-SPME-GC×GC-MS

**Figure 8:**
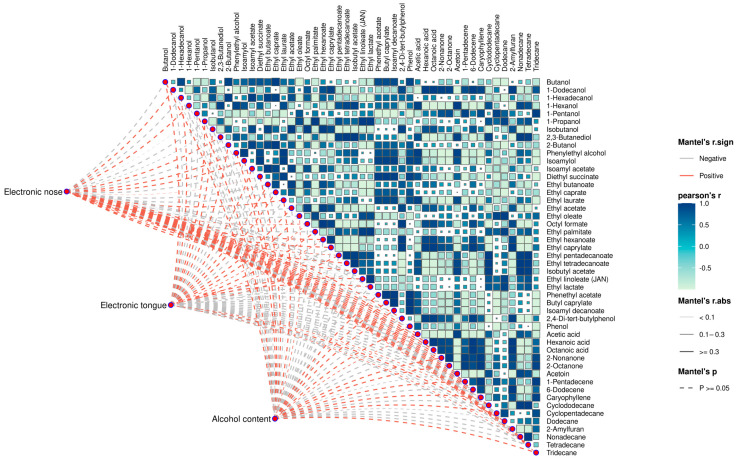
Analysis of the correlation among electronic sense, alcohol content, and volatile compounds. The color in each heatmap grid indicates the positive and negative correlation coefficients between the volatile compounds, while the color block size denotes the absolute value of the correlation coefficient.

Correlations among HS-SPME-GC×GC-MS, E-nose, E-tongue, and alcohol content were assessed using Pearson and Mantel tests. The results revealed strong associations between aroma, taste, alcohol, and VFCs. Figure 8 illustrates the score changes in the *r*-values of the Mantel test between three matrix variables (E-nose, E-tongue, and alcohol content). The E-nose showed a strong positive correlation with 13 compounds (*r* > 0.80), among which acetic acid, 2-nonanone, 1-dodecanol, 1-hexanol, and acetoin had higher correlations (*r* > 0.90). These results indicated that the E-nose could effectively distinguish the flavor characteristics of raw *Baijiu* at each distillation stage. The E-tongue showed strong positive correlations (*r* > 0.90) with eight compounds and strong negative correlations (*r* > −0.90) with five compounds, demonstrating that the taste is also affected by the flavor substances produced by raw *Baijiu*. Alcohol content was positively correlated with nine compounds (*r* > 0.90) and negatively correlated with six compounds (*r* > −0.90), of which ten were alcohol and esters: diethyl succinate, 1-pentanol, phenethyl acetate, butyl caprylate, isoamyl decanoate, isoamylol, isobutanol, ethyl pentadecanoate, ethyl acetate, and octyl formate. This finding indicated that the change in alcohol content during distillation process has a close effect on the production of alcohol and ester compounds. Existing related research presents different trends. Wang et al. showed that alcohol content was negatively correlated with the reduction of esters [9]. Li et al. found that the total ester content of *NXX Baijiu* showed a trend of “decrease-equilibrium-increase-decrease” with a decrease in alcohol content during distillation [9]. For example, ethyl hexanoate was found to be mainly present in high-alcohol liquors during distillation. In our study, ethyl hexanoate was also found to be more abundant in the early stages of distillation than in the middle and later stages of distillation (Table 2, Figure S2). This might be due to the fact that ethyl hexanoate is mainly formed by esterification of hexanoic acid with ethanol and has a high boiling point.

## 4. Conclusions

This study first utilized E-nose and E-tongue combined with HS-SPME-GC×GC-MS technologies to comprehensively analyze the changes in flavor compounds, aroma-active components, and alcohol content across three distillation stages of raw *SQX Baijiu*. These findings revealed that the E-nose and E-tongue effectively characterized the flavor profiles of different distillation stages, with alcohol and ester compounds showing the highest response values. Various compounds exhibited distinct trends, and were affected by the alcohol content. The head *Baijiu* exhibits the most pronounced differences compared with both the heart and tail *Baijiu* in alcohol content and all types of flavor substances. While the differences in varieties of flavor compounds between the heart and tail *Baijiu* were primarily manifested in esters, acids, and other types. The total concentration of volatile compounds gradually decreased from the head to the tail *Baijiu*. The head *Baijiu* contains more abundant flavor compounds than the heart and tail *Baijiu*. Alcohols initially increased and then decreased as distillation progressed, while the concentration of acids gradually increased. In contrast, aldehydes and ketones decreased progressively during distillation. Ethyl caprylate was identified as the most important aroma-active compounds during the distillation of *SQX Baijiu*. Isoamylol effectively differentiated the raw *Baijiu* from the three distillation stages. 1,1-Diethoxyethane, 1-pentanol, 1-propanol, and isoamylol were the main contributors to the overall flavor of the raw *Baijiu*. The combination of E-nose, E-tongue, and HS-SPME-GC×GC-MS technologies integrates the strengths of each method, providing a more comprehensive reflection of flavor profile changes in the distilled *SQX Baijiu*. E-nose and E-tongue quickly and effectively distinguished the flavor characteristics of the raw Baijiu samples. The research results indicate that the production technology of this distillery has reached a relatively excellent level, effectively removing methanol and furfural-like substances from the raw *Baijiu*. Trace amounts of harmful off-flavor substances were only detected in the head *Baijiu.* This characteristic could be utilized to maximize the use of both head and tail *Baijiu* in subsequent production. Furthermore, the process could be further optimized by adjusting rice husk dosage and distillation conditions. In addition, other advanced analytical techniques (such as ultraviolet–visible spectroscopy and infrared spectroscopy) can be employed for verification and in-depth investigation of key compounds (such as 5-hydroxymethylfurfural). This study enhances our understanding of the changes in volatile compounds and their aroma contributions during the distillation process of *SQX Baijiu*. It provides a theoretical basis for optimizing the distillation and classification processes as well as reducing health risk.

## Figures and Tables

**Table 1:** Performance characteristics of E-nose sensor.

No.	Sensor Name	Sensor Characteristics
1	W1C	Aromatic
2	W5S	Nitrogen oxides
3	W3C	Ammonia
4	W6S	Hydrogen
5	W5C	Alkane
6	W1S	Methane
7	W1W	Sulfur
8	W2S	Alcohol and aromatic
9	W2W	Aromatic and sulfur organic
10	W3S	Long-chain alkanes

**Table 2:** Concentration and types of volatile compounds in different distilled raw *Baijiu*. Results are mean values of triplicates (*n* = 3) ± SD.

Compound Name	RI	CAS	Concentration (μg/L)		
			Head	Heart	Tail
**Alcohols**					
Butanol	1157	71-36-3	2049.67 ± 129.44	1803.81 ± 40.46	2337.96 ± 49.12
1-Dodecanol	1457	112-53-8	20,034.05 ± 1038.91	10,259.83 ± 449.5	8081.75 ± 356.05
1-Heptatriacotanol	——	105794-58-9	222.81 ± 15.75	ND	406.55 ± 18.72
1-Hexadecanol	2363	36653-82-4	4266.68 ± 289.23	ND	5030.21 ± 208.72
1-Hexanol	1355	111-27-3	1131.6 ± 47.71	1450.31 ± 77.14	1512.58 ± 99.25
1-Pentanol	1304	71-41-0	212,531.03 ± 7448.53	205,893.75 ± 7651.94	152.13 ± 9.7
1-Propanol	1030	71-23-8	1371.69 ± 46.86	208,957.01 ± 12,108.89	7244.49 ± 448.32
Isobutanol	1069	78-83-1	81,870.28 ± 1031.78	78,017.26 ± 4364.74	80,113.47 ± 5455.88
2,3-Butanediol	32,612	513-85-9	3544.6 ± 64.25	4615.15 ± 147.51	4445.95 ± 246.4
2-Butanol	1012	78-92-2	845.43 ± 54.95	ND	1219.48 ± 75.55
6-Methyl-2-heptanol	1380	4730-22-7	150,899.61 ± 7727.97	ND	ND
2-Hexadecanol	1967	14852-31-4	957.56 ± 54.99	ND	ND
2-Nonanol	1563	628-99-9	855.58 ± 58.12	ND	185.57 ± 13.44
2-Tridecanol	1491	1653-31-2	12,280.25 ± 481.12	ND	ND
7-Octen-2-ol	1063	39546-75-3	57,637.36 ± 2366.94	ND	ND
Ethanol	463	64-17-5	93,343.55 ± 4423.99	113,856.41 ± 4444.18	77,354.99 ± 5196.55
Phenylethyl alcohol	28,900	60-12-8	17,285.48 ± 455.85	18,516.54 ± 672.9	19,646.42 ± 1253.51
Isoamylol	891	123-51-3	ND	64,194.54 ± 3818.44	212,773.54 ± 6106.35
1-Docosanol	1897	661-19-8	ND	323.45 ± 22.45	ND
2-Methyl-1-hexadecanol	1519	2490-48-4	ND	ND	469.61 ± 28.51
3-Methyl-2-butanol	1345	598-75-4	ND	ND	577.35 ± 38.13
1-Pentadecanol	1755	629-76-5	ND	ND	612.2 ± 42.75
**Esters**					
Butyl octyl phthalate	2317	84-78-6	382.54 ± 22.14	ND	ND
2,3-Epoxypropyl acetate	——	6387-89-9	3135.17 ± 77.49	ND	ND
Isoamyl acetate	500	123-92-2	71,850.94 ± 1577.75	ND	68,046.6 ± 2623.86
2-Methylbutyl octanoate	1677	67121-39-5	944.82 ± 8.74	ND	ND
Ethyl linoleate	2521	7619-08-1	10,415.94 ± 659.37	ND	7277.39 ± 494.38
Phenethyl acetate	1814	103-45-7	18,563.79 ± 982.97	16,387.6 ± 688.89	ND
Diethyl succinate	1654	123-25-1	5301.89 ± 304.37	5438.28 ± 289.71	6325.1 ± 240.7
Ethyl butanoate	785	105-54-4	7546.14 ± 317.91	6727.59 ± 280.66	7435.59 ± 246.73
Ethyl caprate	1122	110-38-3	59,921.26 ± 3887.13	50,383.81 ± 762.17	65,323.57 ± 2263.44
Ethyl laurate	640	106-33-2	22,828.21 ± 646.9	25,324.32 ± 426.79	29,034.04 ± 2156.05
Ethyl acetate	870	141-78-6	140,339.3 ± 5054.45	127,374.79 ± 4402.54	135,458.73 ± 4765.65
Ethyl oleate	2483	111-62-6	18,898.89 ± 1213.96	25,330.64 ± 760.86	13,912.74 ± 596.04
Octyl formate	1175	112-32-3	2044.48 ± 77.57	ND	893.48 ± 25.73
Ethyl palmitate	2265	628-97-7	88,693.79 ± 3251.69	128,573.29 ± 7047.21	85,615.13 ± 3015.28
Ethyl hexanoate	1234	123-66-0	20,062.22 ± 901.76	15,398.24 ± 911.38	17,253.17 ± 1170.31
Ethyl caprylate	1430	106-32-1	65,264.5 ± 2000.95	58,036.86 ± 1829.72	59,036.5 ± 3783.13
Ethyl pentadecanoate	2140	41114-00-5	420.79 ± 15.82	946.6 ± 39.27	708.1 ± 22.32
Ethyl DL-leucate	1082	10348-47-7	1043.18 ± 55.45	1285.5 ± 85.26	1355.61 ± 45.8
Ethyl tetradecanoate	2010	124-06-1	4993.16 ± 350.48	8011.68 ± 244.67	7319.21 ± 212.97
Isobutyl acetate	721	110-19-0	ND	3398.14 ± 220.66	2739.43 ± 125.94
Ethyl linoleate (JAN)	2521	544-35-4	ND	13,617.77 ± 682.48	455.53 ± 8.85
Ethyl lactate	1329	97-64-3	ND	40,069.6 ± 1963.73	ND
Diisobutyl phthalate	2548	84-69-5	ND	ND	521.18 ± 21.34
2-Ethylhexyl salicylate	1806	118-60-5	ND	ND	570.93 ± 36.73
Ethyl linolenate	2591	1191-41-9	ND	ND	194.68 ± 8.68
Phenethyl acetate	1814	103-45-7	ND	ND	21,906.64 ± 757.36
Nonyl acetate	1573	143-13-5	ND	ND	314.51 ± 18.53
Pentyl acetate	1167	628-63-7	ND	ND	198.47 ± 12.14
Ethyl 3-phenylpropanoate	1865	2021-28-5	ND	ND	211.65 ± 8.61
Ethyl isopentyl succinate	2219	28024-16-0	ND	ND	272.15 ± 6.25
Amyl butyrate	1321	540-18-1	ND	ND	123.98 ± 3.78
Butyl caprylate	1601	589-75-3	ND	ND	444.94 ± 23
Ethyl heptanoate	1311	106-30-9	ND	ND	56.37 ± 1.95
Ethyl nonadecanoate	2219	18281-04-4	ND	ND	132.51 ± 8.36
Ethyl nonanoate	1547	123-29-5	ND	ND	229.75 ± 11.97
Isopentyl octylate	1417	2035-99-6	ND	ND	1194.9 ± 69.79
Isoamyl decanoate	1864	2306-91-4	ND	ND	795.01 ± 50.45
**Aldehydes**					
5-Hydroxymethylfurfural	2512	67-47-0	123.65 ± 5.23	ND	ND
Acetaldehyde ethyl amyl acetal	1104	13442-89-2	5638.64 ± 335.23	ND	ND
L-glyceraldehyde	——	497-09-6	263.03 ± 13.25	ND	ND
**Phenols**					
2,4-Di-tert-butylphenol	1555	96-76-4	286.6 ± 5.92	ND	74.23 ± 2.89
Phenol	901	108-95-2	1005.17 ± 28.77	905.15 ± 68.51	1927.33 ± 88.06
4-Tert-octylphenol	1631	78721-87-6	ND	ND	2040.04 ± 110.49
o-Hydroxybiphenyl	1515	90-43-7	ND	ND	709.24 ± 34.17
Guaiacol	1862	90-05-1	ND	ND	184.66 ± 13.22
**Acids**					
Linoleic acid	3168	60-33-3	1229.72 ± 87	ND	ND
Acetic acid	1450	64-19-7	23,028.81 ± 1757.85	27463.5 ± 1774.88	30,029.38 ± 2214.89
1,2-Benzenedicarboxylic acid	2037	84-74-2	1178.9 ± 69.47	ND	ND
Hexanoic acid	1843	142-62-1	949.87 ± 51.71	ND	ND
Octanoic acid	1173	124-07-2	697.68 ± 8.28	ND	ND
Formic acid	1987	64-18-6	ND	ND	26.25 ± 1.7
Oleic acid	3172	112-80-1	ND	ND	545.65 ± 16.47
Oxalic acid	1509	144-62-7	ND	ND	851.95 ± 62.33
**Ketones**					
2-Nonanone	1052	821-55-6	1029.15 ± 36.28	739.15 ± 50.76	544.75 ± 23.88
2-Octanone	952	111-13-7	12,602.28 ± 827.61	9661.06 ± 201.98	6909.69 ± 199.85
Acetoin	717	513-86-0	ND	822.37 ± 49.81	982.89 ± 53.78
**Others**					
1-Pentadecene	1502	13360-61-7	12,527.8 ± 891.86	11,636.2 ± 621.84	9912.03 ± 594.14
3-Ethoxy-1-propanol	1371	111-35-3	244.5 ± 16.04	ND	ND
(Z)-6-Dodecene	1240	7206-29-3	5958.58 ± 198.3	ND	ND
7-Tetradecene	1322	10374-74-0	953.38 ± 42.6	ND	ND
Anethole	1817	104-46-1	2570.05 ± 201.92	ND	ND
(1,3-Dimethylbutyl) benzene	1162	19219-84-2	774.72 ± 48.87	ND	ND
(2,2-Diethoxyethyl)-Benzene	1690	6314-97-2	2661.61 ± 85.72	ND	ND
1,2,4-Trimethylbenzene	1288	95-63-6	1571.12 ± 37.16	ND	1794.35 ± 114.42
1-Methyl-2-(1-ethylpropyl) benzene	993	54410-74-1	264 ± 16.8	ND	ND
Hexylbenzene	1291	1077-16-3	1920.58 ± 118.13	ND	1165.61 ± 75.5
Trans-caryophyllene	1494	87-44-5	2303.22 ± 91.6	1992.93 ± 133.14	1670.32 ± 74.7
Cyclododecane	1439	294-62-2	337.13 ± 25.16	9107.21 ± 424.34	5592.45 ± 352.82
Trans-cyclododecene	1558	1486-75-5	630.18 ± 26.05	ND	ND
Cyclopentadecane	1536	295-48-7	14,653.25 ± 680.77	16,304.5 ± 981.7	9856.15 ± 397.16
Dodecane	1214	112-40-3	13,943.3 ± 894.04	15,466.33 ± 985.91	13,187.69 ± 604.86
1,1-Diethoxyethane	894	105-57-7	390,729.32 ± 27,895.13	353,718.47 ± 16,050.45	386,197.22 ± 28,771.85
Formamide	1791	75-12-7	37,292.59 ± 2552.99	ND	ND
2-Amylfuran	1040	3777-69-3	406.89 ± 21.29	ND	ND
Hexadecane	1612	544-76-3	2487.03 ± 61.15	ND	ND
Nonadecane	1910	629-92-5	2055.86 ± 61.3	4041.77 ± 207.29	3536.6 ± 249.44
1,1,3-Triethoxypropane	1201	7789-92-6	1182.08 ± 73.16	ND	ND
Styrene	1254	100-42-5	305.33 ± 11.63	ND	ND
Tetradecane	1413	629-59-4	3927.22 ± 97.45	7684.87 ± 518.41	6129.58 ± 170.05
2,6,10-Trimethyltetradecane	1539	14905-56-7	1373.86 ± 44.86	ND	ND
Tridecane	1313	629-50-5	93,510.88 ± 6521.28	86,483.63 ± 5892.13	62,884.01 ± 4039.01
Cis-3-dodecene	1254	7239-23-8	ND	12,487.76 ± 587.62	ND
Acetic anhydride	1236	108-24-7	ND	ND	222.24 ± 9.56
Coumaran	2389	496-16-2	ND	ND	86.57 ± 2.35
Eicosane	2000	112-95-8	ND	ND	ND
Furfuryl ethyl ether	1272	6270-56-0	ND	ND	48.88 ± 2.74
Heptadecane	1711	629-78-7	ND	ND	1211.61 ± 88.41
Naphthalene	1745	91-20-3	ND	ND	59.17 ± 2.74

Note: “ND” represents that this compound is not detected in samples.

**Table 3:** ROAVs of major aroma compounds (ROAV > 1) in different distilled raw *Baijiu*.

NO.	Aroma Compounds	Odor Thresholds (μg/L)	Descriptor	ROAV		
				Head	Heart	Tail
1	Ethyl butanoate	82 [50]	Pineapple, fruity	92.03	82.04	90.68
2	2-Nonanone	483 [51]	—	2.13	1.53	1.13
3	1-Dodecanol	1000 [52]	Fatty, waxy odor	20.03	10.26	8.08
4	2-Nonanol	58 [53]	Fatty	14.75	—	3.2
5	Ethanol	8800 [54]	Strong alcoholic	10.61	12.94	8.79
6	Phenethyl acetate	407 [55]	Rose	—	—	53.82
7	1-Pentanol	37,400 [56]	Fruity	5.68	5.51	—
8	Isoamyl acetate	500 [48]	Banana, sweet	143.7	—	136.09
9	Ethyl caprate	1120 [26]	Fresh, fruity	53.5	44.99	58.32
10	Ethyl laurate	500 [57]	Sweet, waxy, floral	45.66	50.65	58.07
11	Ethyl acetate	32,600 [26]	Pineapple	4.3	3.91	4.16
12	Ethyl palmitate	4500 [58]	Fruity, creamy	19.71	28.57	19.03
13	Ethyl hexanoate	200 [48]	Fruity, sweet	100.31	76.99	86.27
14	Ethyl caprylate	13 [26]	Fruity, grape	5020.35	4464.37	4541.27
15	Isobutyl acetate	922 [48]	Fruity, rum	—	3.69	2.97
16	Pentyl acetate	70 [56]	Banana	—	—	2.84
17	Ethyl 3-phenylpropanoate	125 [59]	—	—	—	1.69
18	Guaiacol	9.5 [50]	Guaiacol	—	—	19.44
19	1-Pentanol	37,400 [56]	Fruity	5.68	5.51	—
20	2-Octanone	230 [51]	—	54.79	42	30.04
21	Acetoin	259 [58]	Sweet, cream	—	3.18	3.79
22	1,1-Diethoxyethane	2090 [48]	Fruity	186.95	169.24	184.78

## Data Availability

The original contributions presented in the study are included in the article/Supplementary Materials, further inquiries can be directed to the corresponding author.

## Supplementary Materials

The following supporting information can be downloaded at: https://www.mdpi.com/article/10.3390/foods14162814/s1, Table S1. Statistics of volatile compounds which common and unique to the head, heart, and tail *Baijiu*. Table S2. VIP values of differential VFCs in the raw *Baijiu* of different distillation stages. Table S3. VIP values of differential ROAV in different distilled raw *Baijiu*. Table S4. The three-dimensional PCA output data. Table S5: The three-dimensional PCA output data. Figure S1. PLS-DA analysis of aroma-active compounds in head, heart, and tail Baijiu: (A) permutation test, (B) loading plot. Figure S2. Change in ethyl hexanoate concentration during distillation. Note: * indicates *p* < 0.05, ** indicates *p* < 0.01.
