# Supplementary material for: Insights into the Flavor Profiles and Key Aroma-Active Compounds of Sichuan Xiaoqu Qingxiangxing Baijiu Across Distilling Stages
Source: Foods. 2025 Aug 14;14(16):2814. doi: 10.3390/foods14162814 (PMC12385443; doi:10.3390/foods14162814)
Supplement: Supplementary file 1 [file foods-14-02814-s001.zip › foods-3765351-supplementary.pdf]

Supplementary Materials

Table S1 Statistics of volatile compounds which common and unique to the head, heart, tail *Baijiu*.

Stages	Common volatile compounds	Unique volatile compounds
Head	1,1-Diethoxyethane, 1-Pentanol, Ethyl Acetate, Tridecane, Ethanol, Ethyl palmitate, Isobutanol, Ethyl caprylate, Ethyl caprate, Acetic acid, Ethyl laurate, Ethyl hexanoate, 1-Dodecanol, Ethyl Oleate, Phenylethyl Alcohol, Cyclopentadecane, Dodecane, 2-Octanone, 1-Pentadecene, Ethyl butanoate, Diethyl succinate, Ethyl tetradecanoate, Tetradecane, 2,3-Butanediol, Trans-Caryophyllene, Nonadecane, Butanol, 1-Propanol, 1-Hexanol, Ethyl DL-leucate, 2-Nonanone, Phenol, Ethyl pentadecanoate, Cyclododecane	Linoleic acid, 6-Methyl-2-heptanol, 7-Octen-2-ol, Formamide, 2-Tridecanol, (Z)-6-Dodecene, Acetaldehyde ethyl amyl acetal, 2,6,10-trimethyltetradecane, (2,2-Diethoxyethyl)-Benzene, 2,3-epoxypropyl acetate, Anethole, Hexadecane, 1,1,3-Triethoxypropane, Hexanoic acid, 1,2-Benzenedicarboxylic acid, 2-Hexadecanol, 7-Tetradecene, 2-Methylbutyl octanoate, (1,3-Dimethylbutyl) benzene, Octanoic acid, Eicosane, Trans-Cyclododecene, 2-Amylfuran, Butyl octyl phthalate, Styrene, 1-Methyl-2-(1-ethylpropyl) benzene, L-glyceraldehyde, 3-Ethoxy-1-propanol, 5-Hydroxymethylfurfural
Heart		Ethyl lactate, Cis-3-dodecene, 1-Docosanol
Tail		Phenethyl acetate, 4-Tert-octylphenol, Heptadecane, Isopentyl octylate, Oxalic acid, Isoamyl decanoate, O-Hydroxybiphenyl, 1-Pentadecanol, 3-Methyl-2-butanol, 2-Ethylhexyl salicylate, Oleic Acid, Diisobutyl phthalate, 2-Methyl-1-hexadecane, Butyl caprylate, Nonyl acetate, Ethyl isopentyl succinate, Ethyl nonanoate, Acetic anhydride, Ethyl 3-phenylpropanoate, Pentyl acetate, Ethyl linolenate, Guaiacol, Ethyl nonadecanoate, Amyl butyrate, Coumaran, Naphthalene, Ethyl heptanoate, Furfuryl ethyl ether, Formic acid

Table S2 VIP values of differential volatile compounds in different distilled raw *Baijiu.*

Compounds Name	VIP
Isoamylol	3.254305193
Phenethyl acetate	2.600959989
6-Dodecene	2.199723416

1-Pentanol	2.157239934
Isobutyl acetate	1.970407998
Acetoin	1.665252431
Hexanoic acid	1.655815643
Isoamyl decanoate	1.612754352
Octanoic acid	1.564872542
Butyl caprylate	1.440057798
2-Amylfuran	1.404858734
Cyclododecane	0.834193984
Isoamyl acetate	0.578481309
Ethyl lactate	0.546216214
1-Propanol	0.542424504
2,4-Di-tert-butylphenol	0.448344551
1-Hexadecanol	0.424286588
Octyl formate	0.418979806
2-Butanol	0.351540833
1-Dodecanol	0.268069018

Phenol	0.194623602
2-Nonanone	0.188066196
2-Octanone	0.177140183
Nonadecane	0.160747575
Ethyl pentadecanoate	0.156218379
Tetradecane	0.133325596
Cyclopentadecane	0.119013145
Tridecane	0.117553989
Ethyl tetradecanoate	0.114329725
Ethyl linoleate (JAN)	0.110090745
Caryophyllene	0.095045037
Ethyl oleate	0.094669573
1-Hexanol	0.085404832
Acetic acid	0.078460082
Ethyl laurate	0.070381444
1-Pentadecene	0.069125213
2,3-Butanediol	0.066857347

Diethyl succinate	0.052653015
Ethyl hexanoate	0.045958363
Butanol	0.041344455
Phenylethyl alcohol	0.037322691
Ethyl caprylate	0.030225154
Ethyl caprate	0.029179254
Ethyl palmitate	0.025341415
Dodecane	0.018157194
Ethyl acetate	0.011272155
Ethyl butanoate	0.007442486
Isobutanol	0.007297365

Table S3 ROAVs of major aroma compounds (ROAV > 1) in different distilled raw *Baijiu*.

NO.	Aroma compounds	Odor thresholds (μg/L)	Descriptor	ROAV		
				Head	Heart	Tail
1	Ethyl butanoate	82[S1]	Pineapple, Fruity	92.03	82.04	90.68
2	2-Nonanone	483[S2]	—	2.13	1.53	1.13

3	1-Dodecanol	1000[S3]	Fatty, waxy odor	20.03	10.26	8.08
4	2-Nonanol	58[S4]	Fatty	14.75	—	3.2
5	Ethanol	8800[S5]	Strong alcoholic	10.61	12.94	8.79
6	Phenethyl acetate	407[S6]	Rose	—	—	53.82
7	1-Pentanol	37400[s7]	Fruity	5.68	5.51	—
8	Isoamyl acetate	500[S8]	Banana, sweet	143.7	—	136.09
9	Ethyl caprate	1120[S9]	Fresh, fruity	53.5	44.99	58.32
10	Ethyl laurate	500[S10]	Sweet, waxy, floral	45.66	50.65	58.07
11	Ethyl acetate	32600[S9]	Pineapple	4.3	3.91	4.16
12	Ethyl palmitate	4500[S11]	Fruity, creamy	19.71	28.57	19.03
13	Ethyl hexanoate	200[S8]	Fruity, sweet	100.31	76.99	86.27
14	Ethyl caprylate	13[S9]	Fruity, grape	5020.35	4464.37	4541.27
15	Isobutyl acetate	922[S8]	Fruity, rum	—	3.69	2.97
16	Pentyl acetate	70[S7]	Banana	—	—	2.84
17	Ethyl 3-phenylpropanoate	125[S12]	—	—	—	1.69
18	Guaiacol	9.5[S1]	Guaiacol	—	—	19.44

19	1-Pentanol	37400[s7]	Fruity	5.68	5.51	—
20	2-Octanone	230[S2]	—	54.79	42	30.04
21	Acetoin	259[S11]	Sweet, cream	—	3.18	3.79
22	1,1-Diethoxyethane	2090[S8]	Fruity	186.95	169.24	184.78

Thresholds were taken from the references S1- S12:

[S1] Yang, Y., Ai, L., Mu, Z., Liu, H., Yan, X., Ni, L., ... & Xia, Y. (2022). Flavor compounds with high odor activity values (OAV > 1) dominate the aroma of aged Chinese rice wine (Huangjiu) by molecular association. *Food Chemistry*, *383*, 132370. https://doi.org/10.1016/j.foodchem.2022.132370

[S2] Fan, S. S., Guan, G. K., Su, Y. Z., Wan, Z. R., Liu, M. K., Wang, T., ... & Chen, S. (2021). Characterization of the aroma-active components in Lanling Meijiu based on GC-O-MS and OAV. *Food and Fermentation Industries*, 47(1) : 243-249. https://doi.org/10.13995/j.cnki.11-1802/ts.025000

[S3] Mi, Y., Wang, Z., Guan, L., Zhang, M., Li, S., Ye, G., ... & Liang, S. (2023). Analysis of volatile compounds in rice porridge of different japonica rice varieties in Northeast China. *Journal of Cereal Science*, *113*, 103749. https://doi.org/10.1016/j.jcs.2023.103749

[S4] Liu, J., Zhao, W., Li, S., Zhang, A., Zhang, Y., & Liu, S. (2015). Determination of volatile compounds in foxtail millet sake using headspace solid-phase microextraction and gas chromatography-mass spectrometry. *Journal of Chemistry*, 2015. https://doi.org/10.1155/2015/239016

[S5] Gemert, V. (2003). Compilations of flavour threshold values in air, water and other media. *Utrecht: Oliemans Punter & Partners BV, Utrecht.*

[S6] Fan, H., Fan, W., & Xu, Y. (2015). Characterization of Key Odorants in Chinese Chixiang Aroma-Type Liquor by Gas Chromatography-Olfactometry, Quantitative Measurements, Aroma Recombination, and Omission Studies. *Journal of Agricultural and Food Chemistry, 63*(14), 3660–3668. https://doi.org/10.1021/jf506238f.

[S7] Xu, Y. . (2021). Comparison of potent odorants in traditional and modern types of chinese xiaoqu liquor (baijiu) based on odor activity values and multivariate analyses. *Foods*, 10(10), 2392. https://doi.org/10.3390/foods10102392

[S8] Sun, X., Qian, Q., Xiong, Y., Xie, Q., Yue, X., Liu, J., Wei, S., & Yang, Q. (2022). Characterization of the key aroma compounds in aged Chinese Xiaoqu Baijiu by means of the sensomics approach. *Food chemistry*, 384, 132452. https://doi.org/10.1016/j.foodchem.2022.132452

[S9] Liu, Q.-R., Zhang, X.-J., Zheng, L., Meng, L.-J., Liu, G.-Q., Yang, T., Lu, Z.-M., Chai, L. J., Wang, S.-T., Shi, J.-S.,Shen, C.-H., & Xu, Z.-H. (2023). Machine learning based age-authentication assisted by chemo-kinetics: Case study of strong-flavor Chinese Baijiu. *Food Research International*, 167. https://doi.org/10.1016/j.foodres.2023.112594

[S10] Xu, Y., Zhao, J., Liu, X., Zhang, C., Zhao, Z., Li, X., & Sun, B. (2022). Flavor mystery of Chinese traditional fermented baijiu: The great contribution of ester compounds. *Food chemistry*, 369, 130920. https://doi.org/10.1016/j.foodchem.2021.130920

[S11] Wang, Z., Wang, Y., Zhu, T., Wang, J., Huang, M., Wei, J., … Meng, N. (2022). Characterization of the key odorants and their content variation in Niulanshan Baijiu with different storage years using flavor sensory omics analysis. *Food Chemistry*, 376. https://doi.org/10.1016/j.foodchem.2021.131851

[S12] Mu, Y., Huang, J., Zhou, R., Zhang, S., Qin, H., Dong, Y., Wang, C., Wang, X., Pan, Q., & Tang, H. (2023). Comprehensive analysis for the bioturbation effect of space mutation and biofortification on strong-flavor Daqu by high-throughput sequencing, volatile analysis and metabolomics. *Food chemistry*, 403, 134440. https://doi.org/10.1016/j.foodchem.2022.134440

Table S4 VIP values of differential ROAV in different distilled raw *Baijiu.*

Aroma-active compounds	VIP
Ethyl butanoate	3.254305193
2-Nonanone	2.600959989
1-Dodecanol	2.199723416
2-Nonanol	2.157239934
Ethanol	1.970407998
Phenethyl acetate	1.665252431
1-Pentanol	1.655815643
Isoamyl acetate	1.612754352
Ethyl caprate	1.564872542

Ethyl laurate	1.440057798
Ethyl acetate	1.404858734
Ethyl palmitate	0.834193984
Ethyl hexanoate	0.578481309
Ethyl caprylate	0.546216214
Isobutyl acetate	0.542424504
Pentyl acetate	0.448344551
Ethyl 3-phenylpropanoate	0.424286588
Guaiacol	0.418979806
1-Pentanol	0.351540833
2-Octanone	0.268069018
Acetoin	0.194623602
1,1-Diethoxyethane	0.188066196

Table S5. The three-dimensional PCA output data

Stages	PCA1	PCA2	PCA3
Head1	-0.304521319	-0.421554753	0.117794361
Head2	-0.364995735	-0.399129451	0.189902421
Head3	-0.608087595	0.561609235	0.097761427

Heart1	0.039513178	0.114140201	-0.528609023
Heart2	0.061028617	0.113190253	-0.453582898
Heart3	0.173128847	-0.460742858	-0.097578158
Tail1	0.327058809	0.216662158	0.665633581
Tail2	0.466826643	0.027286947	0.041163733
Tail3	0.210048556	0.248538269	-0.032485444

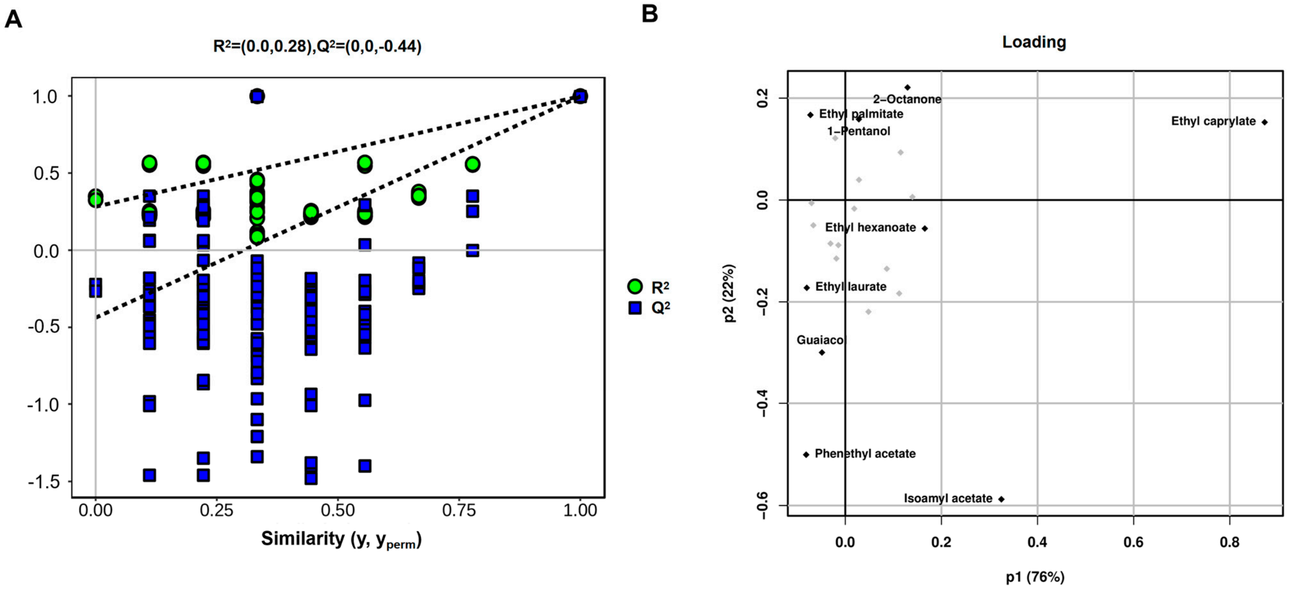

Figure S1. PLS-DA analysis of aroma active compounds in head, heart, tail *Baijiu*: (A) permutation test, (B) loading plot.

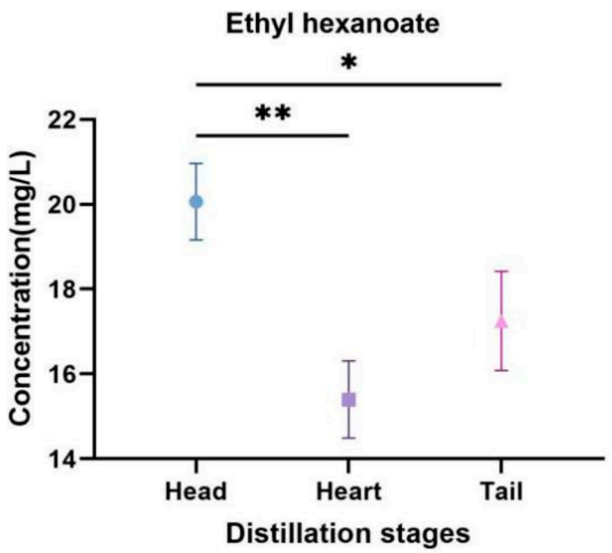

Figure S2. Change of ethyl hexanoate concentration during distillation.

Note: * indicates $p < 0.05$, ** indicates $p < 0.01$.
